# The seroprevalence of several infections in urticaria

**DOI:** 10.1186/1471-2334-14-S7-P72

**Published:** 2014-10-15

**Authors:** Lucia Dinu, Corina Daniela Ene (Nicolae), Mircea Tampa, Lucreția Dulgheru, Dumitru Justin Diaconu

**Affiliations:** 1 Department of Dermatology, MedLife Clinic, Bucharest, Romania; 2 Department of Pharmacology, Carol Davila University of Medicine and Pharmacy, Bucharest, Romania; 3 Department of Dermatology, Carol Davila University of Medicine and Pharmacy, Bucharest, Romania; 4 Department of Dermatology, Clinical Hospital of Infectious and Tropical Diseases “Dr. Victor Babeş”, Bucharest, Romania; 5 "Dr. Victor Babeş" Diagnosis and Treatment Medical Center, Bucharest, Romania

## Background

Over the time, many infections were attributed to be the cause of urticaria. We proposed to assess the seroprevalence of hepatitis B virus (HBV), hepatitis C virus (HCV) and *Helicobacter pylori* infection in acute and chronic spontaneous urticaria patients.

## Methods

We conducted a prospective study which included 236 acute spontaneous urticaria patients, 168 chronic spontaneous urticaria and 220 volunteers without urticaria. The study was approved by the Ethics Committee of the Hospital and all the patients gave their consent for the inclusion into the research protocol.

## Results

Serology for hepatitis B was found to be positive in 8 patients (3.38%) in the acute spontaneous urticaria group, in 9 patients (5.35%) in the chronic spontaneous urticaria group and in 5 subjects (2.27%) in the control group. Antibodies to HCV were identified in 3 patients (1.27%) with acute spontaneous urticaria, in 4 patients with chronic spontaneous urticaria (2.38%) and in one subject (0.45%) in the control group. Antibodies to *Helicobacter pylori* were present in 139 acute spontaneous urticaria patients (58.89%), in 107 chronic spontaneous urticaria patients (63.69%) and in 124 (56.36%) controls.

## Conclusion

Based on these results, we can conclude that the incidence of urticaria is higher in patients suffering from infections, but the analyzed pathogens in this study cannot be considered risk factors for the occurrence of urticaria, although their presence may exacerbate the symptomatology and the evolution of this disease. The therapy of these infections may ameliorate urticaria and the efficacy of the H1 antihistamine treatment.

